# Negative Air Ions Attenuate Nicotine-Induced Vascular Endothelial Dysfunction by Suppressing AP1-Mediated FN1 and SPP1

**DOI:** 10.3390/antiox14070859

**Published:** 2025-07-14

**Authors:** Sha Xiao, Tianjing Wei, Mingyang Xiao, Mingming Shan, Ziqi An, Na Li, Jing Zhou, Shuang Zhao, Xiaobo Lu

**Affiliations:** 1 Key Laboratory of Environmental Stress and Chronic Disease Control and Prevention, Ministry of Education, China Medical University, Shenyang 110122, China; hy0208032@muhn.edu.cn (S.X.); woshiweitian@163.com (T.W.); 20191999@cmu.edu.cn (M.X.); mmshan9602@163.com (M.S.); anziqianziqi@163.com (Z.A.); zhaoshuang@163.com (S.Z.); 2 Department of Toxicology, School of Public Health, China Medical University, Shenyang 110122, China; 3 School of Public Health, Key Laboratory of Tropical Translational Medicine of Ministry of Education, Hainan Medical University, Haikou 571199, China; hy0208047@muhn.edu.cn (N.L.); hy0208035@muhn.edu.cn (J.Z.)

**Keywords:** nicotine, hypertension, negative air ions, vascular endothelial dysfunction, activator protein-1

## Abstract

Nicotine-induced oxidative stress contributes significantly to vascular endothelial dysfunction. While negative air ions (NAIs) demonstrate potential blood-pressure-regulating and antioxidant properties, their mechanistic role remains unclear. This study examined the effects of NAIs against nicotine-induced oxidative damage and vascular endothelial injury in spontaneously hypertensive rats (SHRs). Western blotting was used to detect the expression levels of the α7nAChR/MAPK/AP1 pathway. Transcriptomic sequencing was performed to identify the differentially expressed genes after treatment with nicotine or NAIs. Furthermore, reactive oxygen species (ROS), endothelin-1 (ET-1), and [Ca^2+^]_i_ levels were detected in human aortic endothelial cells (HAECs) treated with nicotine, and the relationship between transcription factor activator protein 1 (AP1) and the target genes was further elucidated through ChIP–qPCR. Nicotine exposure in SHRs elevated blood pressure and induced oxidative damage through α7nAChR/MAPK/AP1 pathway activation, causing endothelial structural disruption. These effects manifested as decreased NO/eNOS and increased ET-1/ET_ab_ expression, while these changes were reversed by NAIs. In HAECs, nicotine impaired proliferation while increasing oxidative stress and [Ca^2+^]_i_ levels. This endothelial damage was markedly attenuated by either NAIs or *fibronectin 1* (*Fn1*)/*secreted phosphoprotein 1* (*Spp1*) knockdown. Mechanistically, we identified AP1 as the transcriptional regulator of *FN1* and *SPP1*. NAIs attenuate nicotine-induced endothelial dysfunction in hypertension by inhibiting AP1-mediated FN1 and SPP1 activation, providing novel insights for smoking-associated cardiovascular risk.

## 1. Introduction

The pathogenesis of hypertension involves complex interactions between environmental exposure, genetic susceptibility, and lifestyle factors [1]. Among modifiable risk factors, cigarette smoking represents a major global contributor to cardiovascular morbidity, with particularly strong associations with hypertension development [2]. Nicotine, the primary addictive component of tobacco, promotes hypertensive progression through multiple mechanisms, including disrupting vascular endothelial function, activating pro-inflammatory adhesion cascades, and initiating sustained vascular inflammation [3,4]. These effects are mediated primarily through nicotine’s action as a selective agonist of nicotinic acetylcholine receptors (nAChRs), particularly the α7nAChR subtype [5]. Emerging evidence positions α7nAChR as a critical modulator of intracellular signaling pathways governing fundamental cellular processes, such as proliferation, apoptosis, and metastatic transformation [6,7].

Reactive oxygen species (ROS) serve as dual regulators in hypertensive disorders, maintaining physiological homeostasis while contributing to pathological progression [8]. In the cardiovascular system, NADPH oxidases (NOXs) constitute the predominant source of regulated ROS production. Vascular endothelial cells (VECs), which form the crucial interface between circulation and tissues, orchestrate multiple physiological functions, including oxygen/nutrient delivery, hemodynamic regulation, immunocyte trafficking, and tissue homeostasis maintenance [9]. Nitric oxide (NO) inactivation by ROSs contributes to endothelial dysfunction in essential hypertension [10]. Despite these established associations, the precise molecular mechanisms underlying smoking-associated endothelial dysfunction in hypertensive patients remain poorly understood.

Activator protein-1 (AP1), a transcription factor composed of JUN and FOS family protein dimers, serves as a master regulator of diverse cellular processes [11]. Chronic tobacco smoke exposure significantly elevates AP1 expression and enhances its transcriptional activity [12]. This activation occurs through mitogen-activated protein kinase (MAPK) cascades, which comprise three evolutionarily conserved subfamilies: (1) ERK (extracellular signal-regulated kinases), which mediate growth factor responses, (2) JNK (c-Jun N-terminal kinases), and (3) p38 MAPK (stress-activated protein kinases), which both respond to inflammatory and stress stimuli [13]. Oxidative stress potently activates these MAPK pathways along with NF-κB signaling, creating a pro-inflammatory feedback loop [14,15]. Notably, nicotine induces AP1 and NF-κB activation through ROS-dependent MAPK stimulation, leading to downstream effects such as matrix metalloproteinase-9 (MMP-9) overexpression [16]. The vascular impact of nicotine extends to enhanced calcium influx via voltage-gated channels, the suppression of calcium-activated potassium channels, and the inhibition of NO/cGMP-dependent vasodilatory pathways [17].

Epidemiological evidence demonstrates that natural forest environments confer significant cardiopulmonary and neuropsychological benefits, such as improved cardiovascular function, stress reduction, and mood enhancement [18]. Negative air ions (NAIs), key bioactive components of such environments, are negatively charged gaseous molecules generated through atmospheric electron attachment [19]. Our previous work systematically reviewed the multisystem biological effects of NAIs, particularly their regulatory roles in cardiovascular and respiratory function, metabolic homeostasis, and emotional regulation [20]. Mechanistic studies reveal that NAIs exert antioxidant effects through multiple pathways, including enhancing superoxide dismutase (SOD) activity, promoting oxidative phosphorylation, and optimizing aerobic metabolism [21]. Notably, Kim et al. demonstrated the capacity of NAIs to mitigate particulate-matter-induced oxidative stress and inflammation via inhibition of the ROS/p38 MAPK/AP1 axis in keratinocytes [22]. This finding acquires particular relevance given nicotine’s established role in ROS generation and MAPK pathway activation. While nicotine promotes oxidative stress and NAIs function as natural antioxidants, their potential interactions in hypertensive pathophysiology remain unexplored, representing a critical gap in our understanding of environmental modulators of cardiovascular health.

This study aimed to elucidate the molecular mechanisms underlying nicotine-induced vascular endothelial dysfunction, with particular focus on the α7nAChR/MAPK/AP1 signaling axis. While direct evidence connecting nicotine and NAIs remains limited, we hypothesized that NAIs may counteract nicotine’s detrimental effects by modulating shared oxidative stress pathways. Our RNA sequencing analysis of the thoracic aorta of spontaneously hypertensive rats (SHRs) identified *fibronectin 1* (*Fn1*) and *secreted phosphoprotein 1* (*Spp1*) as AP1-regulated targets, establishing a potential mechanistic link between nicotine exposure and NAIs intervention. These findings provide novel insights into the pathogenic mechanisms of smoking-associated hypertension and potential environmental preventive strategies for high-risk populations.

## 2. Materials and Methods

### 2.1. Animals and Treatments

All experimental procedures were approved by the Institutional Animal Care and Use Committee of China Medical University (Approval No. CMU2021219) in compliance with national guidelines (National Experimental Animal Use License No. SYXK[Liao]-2018-0008). Male Wistar-Kyoto (WKY, *n* = 20) and spontaneously hypertensive rats (SHRs, *n* = 100) were obtained from Charles River Laboratories (Beijing, China) and maintained under controlled environmental conditions (12-h light/dark cycle, a relative temperature of 25 ± 2 °C, a relative humidity of 50% ± 2%, and free access to food and water). Unless otherwise specified, all experimental protocols followed our previously established methods [23].

Male rats aged 8 weeks were used in the experiments. After one week of adaptive feeding, 20 WKY rats were randomly divided into two groups of 10, with a normal blood pressure control group (WKY) with normal feeding for 1 month or 3 months. Furthermore, 100 SHRs were randomly divided into ten groups: a hypertension model group (SHR), a 0.5 mg/kg nicotine group (the low-dose nicotine group, LNIC SHR), a 1.5 mg/kg nicotine group (the high-dose nicotine group, HNIC SHR), a group receiving 0.5 mg/kg nicotine and 6 h of NAIs per day (the low-dose nicotine intervention group, LNIC+6h NAIs SHR), and a group receiving 1.5 mg/kg nicotine and 6 h of NAIs per day (the high-dose nicotine intervention group, HNIC+6h NAIs SHR).

The nicotine exposure groups received intraperitoneal injections of 0.5 mg/kg or 1.5 mg/kg nicotine (JOT-10378, Pu Fei De biotechnology company, Chengdu, China) administered daily at a consistent time point for five consecutive days per week, continuing for either 1 or 3 months. The selected dosage regimen was based on pharmacokinetic evidence demonstrating that plasma cotinine levels (204~364 ng/mL) following 1 mg/kg nicotine administration in rodents closely approximate concentrations observed in habitual smokers [24]. This validation supports our use of the 0.5 mg/kg (representing moderate exposure) and 1.5 mg/kg (simulating heavy smoking) dose ranges for mechanistic studies.

NAI interventions were conducted using a commercial negative ion generator (A8 Zuo Shan Technology Company, Yantai, China) within a standardized 27 m^3^ exposure chamber (3 m × 3 m × 3 m). NAI concentrations were continuously monitored using a calibrated negative ion detector (KEC-990, KEC Company, Shenzhen, China). The NAI intervention groups were exposed to 4.5 × 10^4^ to 5 × 10^4^ NAIs/cm^3^ (equivalent to five times the concentration of NAIs in natural environments such as forests and coastal environments) for 6 h at a fixed time every day.

Systolic blood pressure (SBP) was measured daily between 08:00 and 10:00 using a non-invasive tail-cuff system (BP2000, Visitech Systems, Allen, TX, USA). Urinary cotinine levels were quantified at 0-, 1-, 2-, and 3-month intervals using a commercial ELISA kit (Jingmei Biological Company, Beijing, China). At the end of the experiment, the rats were anesthetized with chloral hydrate, their chests were quickly cut open, and their organs were removed and weighed (wet weight). Blood samples were collected from the abdominal aorta of rats to determine the serum levels of the oxidative damage index, NO, and ET-1. A portion of the thoracic aorta was fixed in 4% paraformaldehyde and embedded in paraffin after 48 h, and another part of the thoracic aorta was stored in an ultra-low-temperature freezer.

### 2.2. Cell Culture and Transfection

Human aortic endothelial cells (HAECs) were purchased from Se Bi Kang Biotechnology Company (iCell-0015a, Shanghai, China). HAECs were cultured in DMEM supplemented with 1% endothelial cell culture additive (iCell-0015a-001b, Se Bi Kang Biotechnology Company, Shanghai, China), 5% fetal bovine serum, 100 U/mL penicillin, and 100 μg/mL streptomycin. The cells were maintained at 37 °C in a 5% CO_2_ atmosphere, and the medium was changed every two days. Transfections were performed using Lipofectamine 2000 reagent (Thermo, Waltham, MA, USA) according to the manufacturer’s instructions. A total of 5 × 10^5^ cells were transfected with 20 nmol/mL siRNA (Ribobio, Guangzhou, China) in a 6-well plate. The cells were allowed to recover in complete media for 24 h and were then harvested at the indicated times. The knockdown efficiency was assessed by measuring the residual gene expression relative to that in the scramble group using Western blotting. The primers used for siRNA are listed in Table S1.

### 2.3. Cell Viability Assay

Cells were seeded at a density of 5000 cells per well in 96-well plates and incubated for 24 h. Then, the cells were treated with 0, 0.1, 0.5, 1, 5, 10, or 20 μM nicotine. After 24 h and 48 h, CCK8 solution (10 µL) was added to each well and incubated for 2 h at 37 °C. The absorbance of each well was determined using a microplate reader (Tecan, Shanghai, China).

The effects of nicotine (0, 0.1, 0.5, 1, 5, 10, and 20 μM) on the viability of HAECs were evaluated (Figure S1). After exposure to 20 μM nicotine for 24 h or 48 h, cell viability decreased to 66.5% and 59.4%, respectively, showing significant cytotoxic effects. After exposure to 0.5 μM and 5 μM nicotine for 24 h or 48 h, cell viability decreased to 95.5%, 86.5%, 90.3%, and 72.4%, respectively. Therefore, we selected 0.5 μM and 5 μM nicotine for the subsequent experiments. The effects of nicotine on the [Ca^2+^]_i_ concentrations and intracellular ROS levels in HAECs were measured by flow cytometry.

### 2.4. Histopathological Analysis

Thoracic aorta tissue was fixed in 4% paraformaldehyde, embedded in paraffin, and sliced into 4 μm thick sections. The sections were stained with hematoxylin–eosin (HE), Verhoeff’s van Gieson (EVG), or dihydroethidium (DHE) and observed under a light microscope.

### 2.5. Oxidative Damage Index, NO, and ET-1 Assays

The serum 8-hydroxylated deoxyguanosine (8-OHdG), SOD, and ET-1 levels of the rats and the ET-1 concentrations in the HAEC supernatants were measured using ELISA kits according to the manufacturer’s protocols (Nanjing Jiancheng Bioengineering Institute, Nanjing, China). A colorimetric method was used to determine the serum MDA (malondialdehyde), SOD, GSH (glutathione), GSSG (glutathione disulfide), and NO levels of the rats. A blood ROS test kit (BestBio, Shanghai, China) was used to examine the blood ROS levels of rats.

### 2.6. RT–PCR

Total RNA was extracted from fresh thoracic aorta tissue or cells using TRIzol reagent (Sigma, St. Louis, MO, USA), and 1 µg of total RNA was reverse-transcribed using 1st Strand cDNA Synthesis SuperMix (TaKaRa RR047A, Dalian, China). Specific synthetic primers and a TaKaRa RR820A PCR kit were used for RT–PCR. The Ct values obtained from the different samples were compared using the 2^−ΔΔCt^ method, and *β-actin* and *GAPDH* served as internal reference genes. The primers used for RT–PCR are listed in Tables S2 and S3.

### 2.7. Western Blotting

Thoracic aorta tissue (0.03 g) or cells were rinsed with PBS, and 300 μL of protein lysis buffer (lysis buffer:PMSF = 100:1) was added. A homogenizer was used to lyse the tissue or cells on ice for 30 min, after which the samples were centrifuged at 12,000 r/min for 20 min at 4 °C. The supernatant containing the extracted protein was collected. The protein concentration was quantified using the BCA method. Twenty micrograms of protein was electrophoresed on 10% SDS polyacrylamide gels and then transferred to polyvinyl difluoride membranes. The membranes were blocked with 5% skim milk at room temperature for 1 h and then incubated with primary antibodies at 4 °C overnight. The membranes were then incubated with secondary antibodies conjugated to horseradish peroxidase. Finally, the immunoreactive bands of the proteins of interest were visualized with the High-sig ECL Western Blotting Substrate (Tanon, Shanghai, China) and then measured using ImageJ software, version 1.53c to quantify protein levels, which were ultimately normalized to β-actin or GAPDH.

Antibodies against α7nAChR (1:1000 dilution) and p-JUN (1:1000 dilution) were purchased from Zenbio (Chengdu, China). NOX4 (1:1000 dilution), NOX2 (1:2000 dilution), p38 MAPK (1:1000 dilution), ERK1/2 (1:2000 dilution), JNK (1:2000 dilution), JUN (1:1000 dilution), FN1 (1:500 dilution), SPP1 (1:1000 dilution), β-actin (1:10,000 dilution), and GAPDH (1:10,000 dilution) antibodies were purchased from Proteintech (Wuhan, China). p-p38 MAPK (1:1000 dilution), p-JNK (1:1000 dilution), eNOS (1:1000 dilution), and ET_ab_ (1:1000 dilution) antibodies were purchased from Affinity (Newport Beach, CA, USA). p-ERK1/2 (1:1000 dilution) antibodies were purchased from CST (Danvers, MA, USA).

### 2.8. RNA-Seq and Data Processing

#### 2.8.1. RNA Isolation

Total RNA was extracted from the thoracic aorta tissue according to the manufacturer’s instructions. The RNA concentration and integrity were assessed using a Thermo Scientific NanoDrop 2000 (Hudson, MA, USA).

#### 2.8.2. RNA-Seq Library Construction and Sequencing

Total RNA was used to construct an RNA-seq library. Briefly, mRNA with a polyA structure in total RNA was enriched by oligo (dT) magnetic beads, and the RNA was broken into fragments of approximately 300 bp in length by ion interruption. Using the RNA as a template, 6-base random primers and reverse transcriptase were used to synthesize first-strand cDNA, and second-strand cDNA was synthesized using the first-strand cDNA as a template. After the library was constructed, PCR amplification was used to enrich the library fragments, and then the library was selected according to the fragment size; the library size was 450 bp. Then, the library was inspected with an Agilent 2100 Bioanalyzer (Santa Clara, CA, USA), and its total concentration was analyzed. It was then uniformly diluted to 2 nM, and a single-strand library was formed through alkali denaturation. The library was paired-end (PE)-sequenced using next-generation sequencing (NGS) on the Illumina sequencing platform.

#### 2.8.3. RNA-Seq Data Analysis

Sequencing reads were obtained by the sequencing platform’s recognition of the image data. Fastq-formatted raw data were filtered through in-house Perl scripts to remove reads containing adapters or N bases, as well as low-quality reads, followed by alignment to the rat reference genome using Hisat2. Statistical analysis of the differentiation comparison of read counts was conducted using the DESeq2 package in the R software, version 4.4.1. GO, KEGG, and GSEA analyses of differentially expressed genes were performed with the R package “Cluster Profiler”. The MSigDB database was used for GSEA (https://www.gsea-msigdb.org/gsea/index.jsp, accessed on 1 July 2023). A *p*-value of <0.05 was considered significant.

### 2.9. ChIP–qPCR

Formaldehyde was used to treat HAECs and crosslink the DNA with DNA-binding proteins. Glycine was added to stop the crosslinking. The cells were suspended in 500 μL of cell lysis buffer, and the cells were lysed via ultrasonication. The supernatant was transferred to a new EP tube and centrifuged at 12,000× *g* at 4 °C for 10 min. An antibody (Fn1, Spp1, or IgG) was added to the magnetic beads. After washing and eluting the product, it was heated at 65 °C for 2 h and 95 °C for 10 min to reverse the crosslinking. The qPCR primers were used to amplify sections of the genomic DNA of interest, and EnTurbo™ SYBR Green PCR SuperMix (ELK Biotechnology, EQ001, Wuhan, China) was used to measure the enrichment of the sample relative to the input (% input). The sequences of the Fn1 and Spp1 promoters were downloaded from the UCSC website; the primers used were designed and synthesized by Heng Yi Sai Biotechnology Company (Wuhan, China) and are listed in Table S4.

### 2.10. Statistical Analysis

Data are presented as the mean ± standard error (SE). For quantitative variables, the Shapiro–Wilk test was utilized to assess the normality of the distribution, and an F test was used to examine the homogeneity of variances among groups. Analysis of variance (ANOVA) was used in cases where the data from multiple samples followed a normal distribution and exhibited homogeneity of variance. For data that did not meet these criteria, nonparametric tests were used. Pairwise comparisons were conducted using the LSD test or Dunnett’s *t* test. Each experiment was an independent sample and was repeated three times. All statistical analyses were performed using SPSS (version 22.0), and the GraphPad Prism 8.0 software was used to create the graphs. *p* < 0.05 indicated statistical significance.

## 3. Results

### 3.1. NAIs Attenuate Nicotine-Aggravated Increases in SBP in SHRs

**Figure 1:**
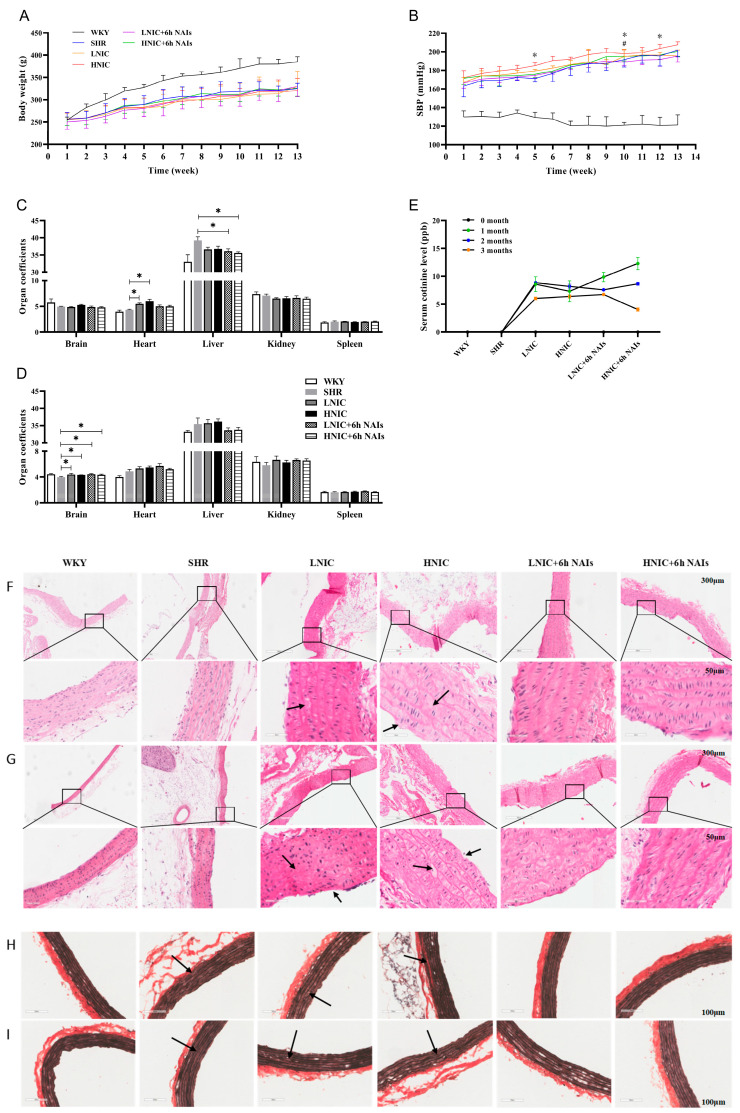
Physiological parameters of rats. (**A**) Body weight (*n* = 10). (**B**) Systolic blood pressure (*n* = 10), * LNIC + 6h NAIs compared with LNIC, *p* < 0.05; # HNIC + 6h NAIs compared with HNIC, *p* < 0.05. Rat organ coefficients (*n* = 5): (**C**) 1 month; (**D**) 3 months. (**E**) Serum cotinine levels (*n* = 3). Effects of nicotine exposure and NAI intervention on thoracic aorta pathology in rats. HE staining: (**F**) 1 month; (**G**) 3 months. Black arrow: endothelial cell defect, thickening of the middle layer, rupture of the smooth muscle fiber layer, and formation of cavities in smooth muscle cells; rupture of the outer membrane. EVG staining: (**H**) 1 month; (**I**) 3 months. Black arrow: the elastic fibers show signs of fracture, loss and degradation. ANOVA, * *p* < 0.05.

Dynamic changes in body weight and SBP in SHRs showed an increasing trend with time (Figure 1A,B). After exposure to nicotine with or without NAI intervention, the SBP of SHRs significantly increased (*p* < 0.05). NAI intervention significantly reduced SBP in SHRs exposed to low-dose nicotine at the 5th, 10th, and 12th weeks, and the effect was most pronounced at the 10th week in those exposed to high-dose nicotine (Figure 1B). In the first month, compared with the SHR group, the heart coefficient in the LNIC and HNIC groups was significantly increased after nicotine treatment, and the liver coefficient in the LNIC+6h NAI and HNIC+6h NAI groups was significantly reduced after nicotine treatment (*p* < 0.05) (Figure 1C). In the third month, only the brain coefficient was significantly increased after nicotine treatment compared with the SHR group (*p* < 0.05) (Figure 1D). Biomarker analysis showed that nicotine significantly increased urinary cotinine levels, though the concentrations exhibited a time-dependent decline (Figure 1E). Partial findings were previously reported in [23].

### 3.2. NAIs Alleviate Nicotine-Induced Thoracic Aortic Injury

Histopathological analysis of nicotine-exposed thoracic aortas revealed significant vascular damage through HE and EVG staining. HE staining demonstrated structural disorganization characterized by intimal layer disruption with endothelial cell loss, medial thickening accompanied by smooth muscle fiber disarray and cytoplasmic vacuolization, and adventitial degradation with extracellular deposits (black arrow in Figure 1F,G). Corresponding EVG staining showed severe elastin pathology, including fiber fragmentation, depletion, and loss of characteristic undulating morphology (black arrow in Figure 1H,I). Notably, the NAI intervention effectively mitigated these nicotine-induced histopathological alterations.

### 3.3. NAIs Reduced Nicotine-Induced Oxidative Damage Through α7nAChR

**Figure 2:**
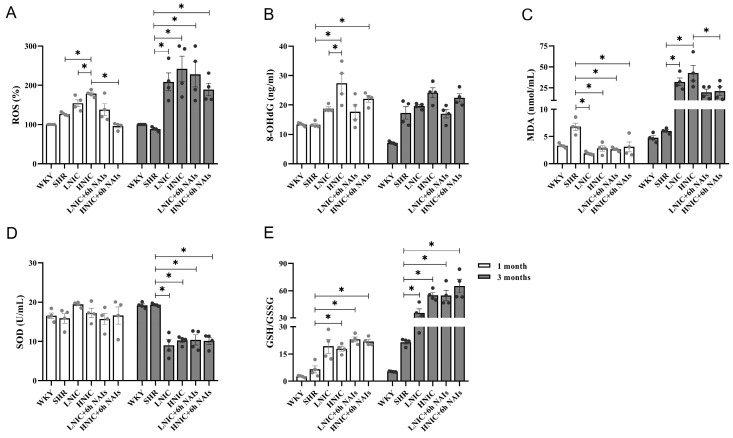

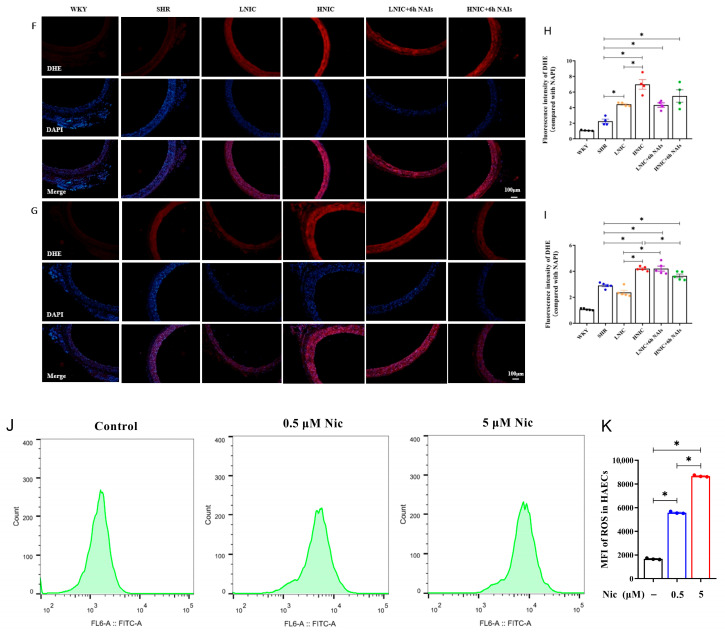
Serum oxidative levels in rats (*n* = 4). (**A**) ROS levels. (**B**) 8-OHdG levels. (**C**) MDA levels. (**D**) SOD levels. (**E**) GSH/GSSG levels. DHE fluorescence staining of thoracic aortas in rats exposed to nicotine (*n* = 4, 100 μm): (**F**) 1 month; (**G**) 3 months. ROS levels in the thoracic aortas of rats: (**H**) 1 month; (**I**) 3 months. (**J**) ROS levels of HAECs treated with nicotine. (**K**) Fluorescence intensity of ROS concentration in HAECs. *p*-values were obtained through ANOVA; * *p* < 0.05.

To evaluate nicotine-induced oxidative damage, we quantified systemic oxidative stress markers (ROS, 8-OHdG, MDA, SOD, and GSH/GSSG) in rats. NAI intervention significantly attenuated the nicotine-induced increase in these oxidative damage indicators (Figure 2A–E). DHE fluorescence staining of thoracic aortas revealed a dose-dependent elevation in ROSs in the SHR, LNIC, and HNIC groups compared to the WKY group at the first month (*p* < 0.05). At the third month, the HNIC group showed significantly higher aortic ROS levels than the SHR controls (*p* < 0.05), while the HNIC+6h NAI intervention markedly reduced ROS accumulation (*p* < 0.05) (Figure 2F–I). In vitro validation in HAECs demonstrated that 24 h of nicotine treatment significantly increased ROS levels (*p* < 0.05) (Figure 2J,K), further confirming the pro-oxidative effects of nicotine.

**Figure 3:**
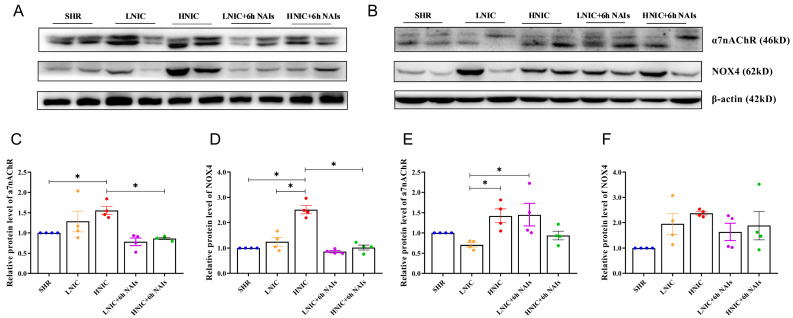

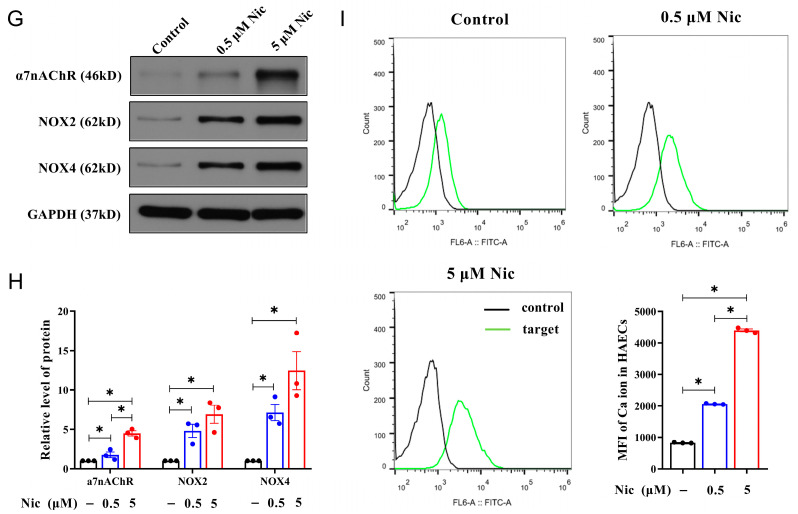
Protein expression of α7nAChR and NOX4 in the thoracic aorta of rats (*n* = 4): (**A**) 1 month; (**B**) 3 months. Protein quantification of α7nAChR and NOX4: (**C**,**D**) 1 month; (**E**,**F**) 3 months. (**G**) Protein expression profiling of α7nAChR, NOX2, and NOX4 in HAECs. (**H**) Protein quantification of α7nAChR, NOX2, and NOX4 in HAECs. (**I**) [Ca^2+^]_i_ concentration of HAECs treated with nicotine; fluorescence intensity at 488 nm. *p* value was obtained by ANOVA; * *p* < 0.05.

The α7nAChR represents the most critical functional subtype in VECs for mediating the effects of nicotine. As NADPH serves as an essential cofactor for both glutathione reduction and ROS generation, and given that NOX2 and NOX4 subunits constitute the primary source of ROSs in the vascular wall, we subsequently investigated the relationship between α7nAChR, NOX4, and nicotine-induced oxidative damage levels (Figure 3A,B). Consistent with our hypothesis, nicotine dose-dependently upregulated α7nAChR and NOX4 protein expression in the thoracic aortas of rats. Notably, the NAI intervention significantly reduced α7nAChR and NOX4 levels at the first month (*p* < 0.05) (Figure 3C,D). While this downward trend persisted at the third month, the differences did not reach statistical significance (Figure 3E,F). Parallel in vitro experiments in HAECs demonstrated similar dose-dependent nicotine-induced upregulation of α7nAChR, NOX2, and NOX4 expression (Figure 3G,H). Flow-cytometric analysis revealed that nicotine significantly increased intracellular calcium concentration in HAECs (*p* < 0.05) (Figure 3I), suggesting α7nAChR-mediated calcium influx as a potential mechanism. Collectively, these results indicate that the effects of nicotine on endothelial function damage are mediated by a7nAChR. NAI intervention attenuated the nicotine-induced oxidative stress.

### 3.4. Nicotine Exposure Reduces NO and Elevates ET-1 Levels in Rat Serum

**Figure 4:**
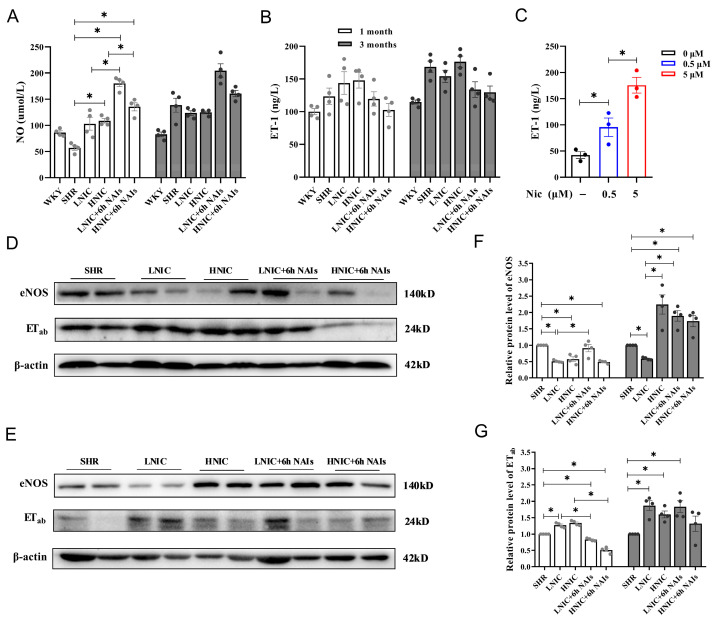
Effects of nicotine exposure and NAI intervention on the function of the vascular endothelium in rats (*n* = 4): (**A**) Serum NO level. (**B**) Serum ET-1 levels. (**C**) Supernatant ET-1 concentration of HAECs treated with nicotine. Protein expression of eNOS and ET_ab_ in thoracic aortas that were exposed to nicotine: (**D**) 1 month; (**E**) 3 months. (**F**,**G**) Protein quantification of eNOS and ET_ab_. *p*-values were obtained through ANOVA; * *p* < 0.05.

We measured serum NO levels using colorimetry and serum ET-1 levels using ELISA. At the first month, both the LNIC and HNIC groups showed modest increases in serum NO compared to SHR controls, while the LNIC+6h NAI and HNIC+6h NAI groups exhibited significant elevations (*p* < 0.05). This trend persisted at 3 months, though without statistical significance (Figure 4A). Conversely, serum ET-1 levels were elevated in the nicotine-treated groups compared to the SHR group but were reduced following NAI intervention (Figure 4B). In vitro validation demonstrated that nicotine significantly increased ET-1 secretion in HAEC supernatants after 24 h of exposure (*p* < 0.05) (Figure 4C). Collectively, these findings indicate that nicotine exposure alters VEC secretion of vasoactive mediators (NO and ET-1), while NAI intervention can counteract these effects.

To comprehensively evaluate vascular endothelial injury, the protein expression of the ET_ab_ receptor and eNOS, which can regulate the levels of NO and ET-1, was detected through Western blotting (Figure 4D,E). At the first month, thoracic aortic eNOS expression was significantly reduced in nicotine-treated groups versus the SHR group (*p* < 0.05), while the LNIC+6h NAI group showed a significant increasing trend (*p* < 0.05) (Figure 4F). At the third month, the ET_ab_ expression was markedly upregulated in nicotine-treated groups compared to the SHR group (*p* < 0.05), an effect significantly attenuated by NAI intervention (*p* < 0.05) (Figure 4G). These results further indicated that nicotine disrupts vascular homeostasis by upregulating the ET_ab_ receptor and downregulating eNOS expression in the thoracic aortas of SHR, consequently altering the systemic NO/ET-1 balance.

### 3.5. Nicotine Exposure Activates the JNK and JUN Signaling Pathways

**Figure 5:**
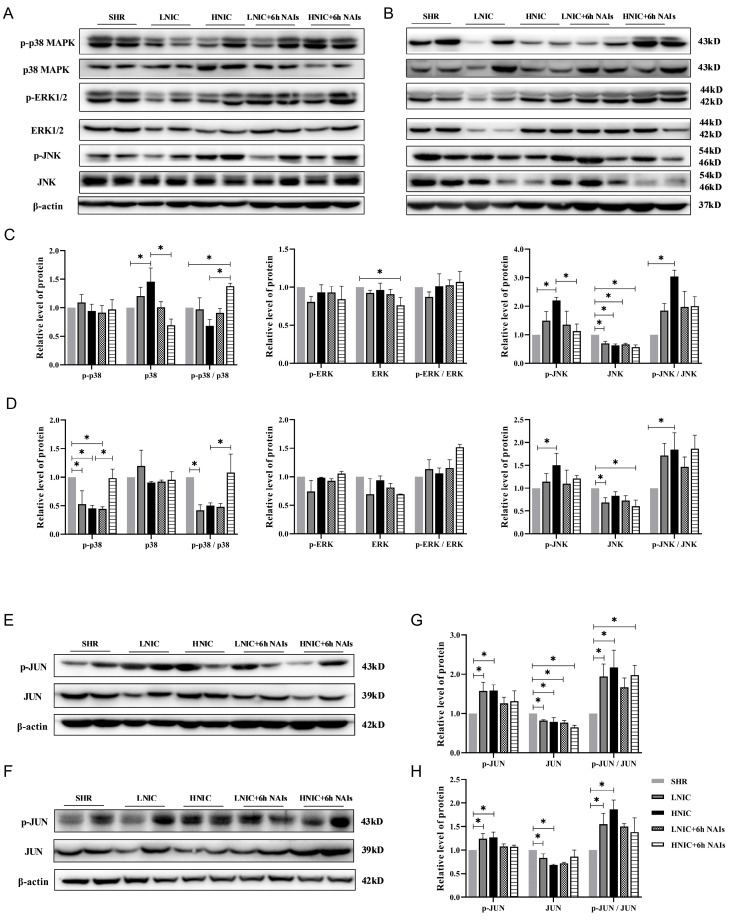
Nicotine exposure promotes the phosphorylation of JNK and JUN in the thoracic aorta of rats (*n* = 4). Protein expression profiling of MAPK pathway genes: (**A**) 1 month; (**B**) 3 months. Protein quantification of MAPK pathway genes in the thoracic aortas of rats: (**C**) 1 month; (**D**) 3 months. Protein expression profiling of JUN in the thoracic aortas of rats: (**E**) 1 month; (**F**) 3 months. Protein quantification of JUN in the thoracic aortas of rats: (**G**) 1 month; (**H**) 3 months. *p*-values were obtained through ANOVA; * *p* < 0.05.

To determine whether nicotine activates the MAPK/AP1 signaling pathway, we analyzed the protein expression of key pathway components in the thoracic aortas of rats using Western blotting. Nicotine treatment dose-dependently increased JNK phosphorylation (p-JNK) levels compared to the controls, with the most pronounced effect being observed in the HNIC group (*p* < 0.05) (Figure 5A–D). A similar activation pattern was observed for JUN phosphorylation (p-JUN) (Figure 5E–H). While NAI treatment showed a tendency to attenuate nicotine-induced phosphorylation of both JNK and JUN, these modulatory effects did not achieve statistical significance.

### 3.6. Exploration of Differentially Expressed Genes in the Thoracic Aortas of Rats Treated with Nicotine and NAIs Using RNA-Seq

**Figure 6:**
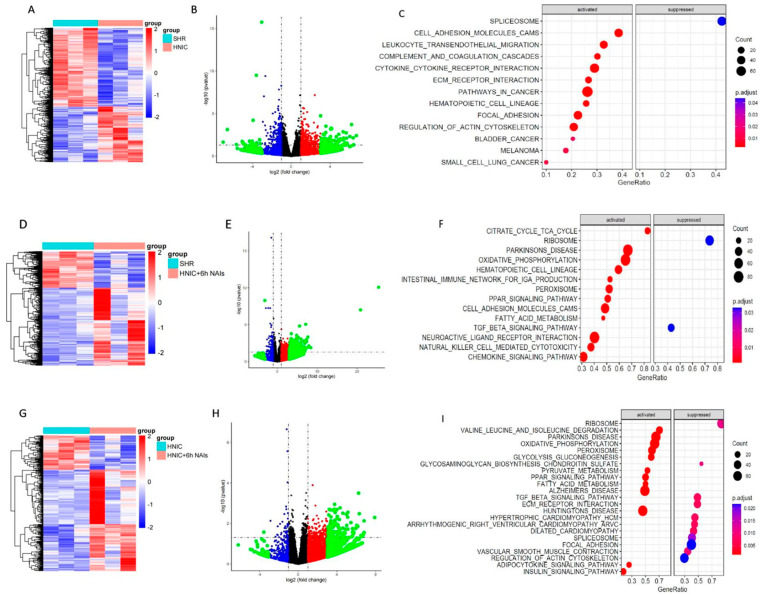
RNA-seq was conducted to explore the regulation of gene expression in the thoracic aorta of rats through nicotine exposure and NAI intervention (*n* = 3). (**A**–**C**) HNIC group compared to the SHR group. (**D**–**F**) HNIC+6h NAI group compared to the SHR group. (**G**–**I**) HNIC+6h NAI group compared to the HNIC group.

To identify key genes responsive to nicotine exposure and NAI intervention, we conducted RNA-seq analysis of differentially expressed genes (DEGs) in thoracic aortas of rats from the SHR, HNIC, and HNIC+6h NAI groups at the third month. Comparative analysis revealed 1342 DEGs in the HNIC group and 1023 DEGs in the HNIC+6h NAI group versus the SHR controls. The HNIC group showed 526 upregulated (41.88%) and 780 downregulated (58.12%) genes, while the HNIC+6h NAI group exhibited 665 upregulated (65.00%) and 358 downregulated (35.00%) genes. Notably, we identified 520 DEGs between the HNIC+6h NAI and HNIC groups, with 380 (73.08%) upregulated and 140 (26.92%) downregulated genes. DEG visualization through volcano plots and hierarchical clustering analysis (performed using R’s Pheatmap package, version 4.4.1) revealed distinct expression patterns among the experimental groups (Figure 6A,D,G). Volcano plots visualized the distribution of DEGs across experimental groups (Figure 6B,E,H). Subsequent KEGG and GSEA-KEGG enrichment analyses revealed distinct pathway alterations. Compared to the SHR group, DEGs in the HNIC group were primarily associated with actin cytoskeleton regulation, focal adhesion, and ECM–receptor interactions (Figure 6C). In contrast, the HNIC+6h NAI group showed upregulation of genes enriched in the chemokine signaling pathway, fatty acid metabolism, cell adhesion molecules, PPAR signaling pathway, and oxidative phosphorylation (Figure 6F). Comparative analysis revealed that downregulated genes in the HNIC+6h NAI group (vs. HNIC group) were predominantly associated with cytoskeletal organization, vascular smooth muscle contraction, focal adhesion, ECM–receptor interactions, and TGF-β signaling (Figure 6I). These findings demonstrate that nicotine exposure upregulates while NAI intervention downregulates genes primarily involved in cytoskeletal signaling, cell–matrix adhesion, and ECM–receptor pathways. Based on these results, we selected the top five candidate genes from these pathways for further investigation: Fn1, Lamc3, Vcam1, Icam1, and Spp1.

Using the JASPAR database, we predicted potential *AP1* transcription factor binding sites within the promoter regions of the Fn1, Lamc3, Vcam1, Icam1, and Spp1 genes (Tables S5–S9). These bioinformatic analyses suggest that AP1 may transcriptionally regulate these genes by directly binding to their promoters, thereby modulating their expression at both the mRNA and protein levels.

### 3.7. FN1 and SPP1 Are Regulated by AP1 and Involved in NAIs Alleviating Nicotine-Induced Damage

**Figure 7:**
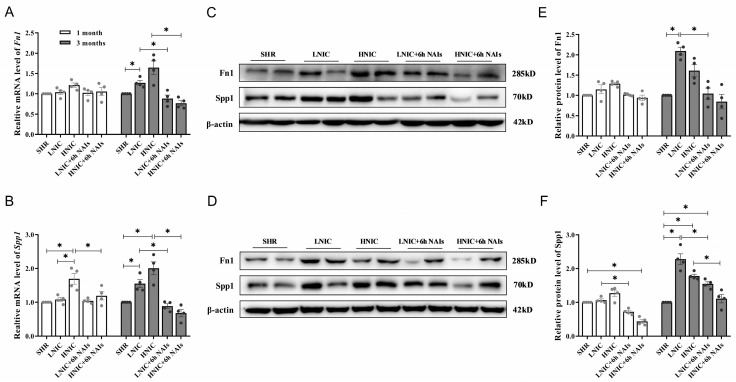

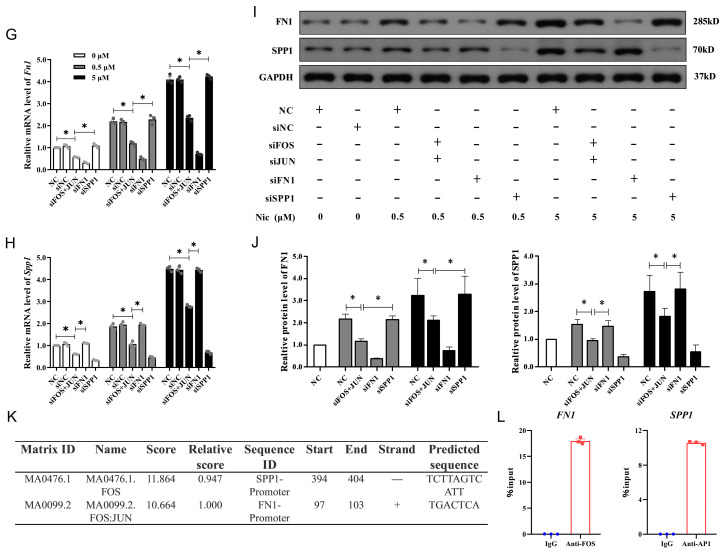
Nicotine upregulates mRNA and protein expression levels of Fn1 and Spp1 in the thoracic aortas of rats (*n* = 4). (**A**) mRNA expression profiling of *Fn1* in rats. (**B**) mRNA expression profiling of *Spp1* in rats. Protein expression profiling of Fn1 and Spp1 in rats: (**C**) 1 month; (**D**) 3 months. Protein quantification of Fn1 and Spp1 in rats: (**E**) 1 month; (**F**) 3 months. Nicotine regulates activation of FN1 and SPP1 in HAECs via AP1 (*n* = 3). (**G**) mRNA expression profiling of *FN1* in HAECs. (**H**) mRNA expression profiling of *SPP1* in HAECs. (**I**) Protein expression profiling of FN1 and SPP1 in HAECs. (**J**) Protein quantification of FN1 and SPP1 in HAECs. (**K**) Candidate putative sequences required for the binding of AP1 to the *FN1* or *SPP1* gene promoter, predicted by the UCSC database and JASPAR database. (**L**) ChIP-qPCR data for FOS binding at the *FN1* promoter and AP1 binding at the *SPP1* promoter in HAECs. *p*-values were obtained through ANOVA; * *p* < 0.05.

To validate the transcriptome sequencing results, we analyzed the mRNA expression of *Fn1* and *Spp1* in the thoracic aortas of rats using qRT–PCR. The qRT–PCR data confirmed the transcriptome findings, demonstrating strong concordance and supporting the reliability of the sequencing results. Specifically, nicotine exposure upregulated Fn1 and Spp1 expression in SHR compared with the untreated SHR group, whereas NAI intervention reversed this effect. At the first month, Spp1 was significantly downregulated following NAI treatment (*p* < 0.05) (Figure 7A). At the third month, both *Fn1* and *Spp1* exhibited marked downregulation upon NAI intervention (*p* < 0.05) (Figure 7B).

Further, we examined the relative protein expression levels of Fn1 and Spp1 in the thoracic aortas of rats via Western blotting (Figure 7C–F). At the first month, the protein expression levels of Fn1 and Spp1 were increased in all groups compared with the SHR group. However, NAI intervention significantly reduced Spp1 expression (*p* < 0.05). At the third month, Fn1 and Spp1 levels were further upregulated in all groups, but NAI treatment again markedly suppressed their expression (*p* < 0.05).

In vitro experiments revealed that nicotine dose-dependently upregulated both the mRNA and protein expression of FN1 and SPP1 in HAECs. Upon AP1 knockdown, the expression levels of FN1 and SPP1 were significantly reduced (*p* < 0.05) (Figure 7G–J). ChIP–qPCR further demonstrated that FOS (an AP1 family member) binds to the FN1 promoter, while AP1 itself directly targets the SPP1 promoter (Figure 7K,L). Collectively, these findings suggest that FN1 and SPP1 contribute to nicotine-induced vascular endothelial dysfunction, with their transcriptional regulation mediated by AP1 family transcription factors.

## 4. Discussion

Tobacco remains the second most prevalent psychoactive substance globally, with current estimates indicating over 1 billion active smokers worldwide [25]. Epidemiological studies consistently identify smoking as an independent risk factor for hypertension, as it is capable of inducing acute elevations in both blood pressure and heart rate [26]. The amount of nicotine in the blood of regular smokers is approximately 220 nmol/L. After inhalation of smoke from a single cigarette, the concentration of nicotine in the blood can reach 440 nmol/L [27]. Despite these well-characterized cardiovascular effects, few therapeutic interventions currently exist that specifically target nicotine-induced vascular endothelial dysfunction and its associated pathological sequelae [28].

Recent evidence indicates that exposure to high-quality natural environments confers cardiovascular benefits, including blood pressure reduction and decreased cardiovascular disease risk. As a key bioactive component of such environments, NAIs have demonstrated therapeutic potential in preclinical studies. Specifically, controlled experiments revealed that adult male Wistar rats exposed to 5000~8000 NAIs/cm^3^ exhibited significant reductions in both SBP and heart rate compared to control groups [29]. Therefore, in the present study, we established a nicotine exposure model using male SHRs to investigate both the detrimental effects of nicotine on vascular endothelium and the protective potential of NAI intervention. Our findings reveal, for the first time, that nicotine impairs vascular endothelial function through the activation of the MAPK/AP1 signaling pathway. Importantly, NAI intervention was found to mitigate nicotine-induced oxidative damage by modulating FN1 and SPP1 expression. In vitro, we have found that nicotine exposure significantly increased oxidative stress in HAECs. The underlying mechanisms involve the upregulation of ET-1 and α7nAChR expression, inhibition of eNOS activity, activation of the MAPK/AP1 pathway through phosphorylation, and induction of FN1 and SPP1 expression mediated by AP1 transcription factor family members.

Accumulating evidence demonstrates that nicotine exposure promotes vascular endothelial dysfunction through oxidative-stress-mediated mechanisms [30], which are mainly characterized by increased oxidase activity of NADPH, the accumulation of lipid peroxides, and decreased activity of antioxidant enzymes [31]. Zhang et al. [32] reported that nicotine exposure could induce vascular endothelial cell injury, aberrant endothelium–monocyte adhesion, platelet aggregation, and vasoconstriction and disrupt vascular NO signaling. Oxidative stress is often associated with the occurrence and development of hypertension. Our results supported that nicotine exposure could aggravate vascular oxidative damage in SHRs, causing pathological vascular changes and impaired elastic fiber function. However, NAI intervention could reduce the oxidation induced by nicotine exposure, indicating that NAIs exert antioxidant effects. Liu et al. [33] demonstrated that elevated concentrations of NAIs ameliorate PM-induced respiratory dysfunction by increasing energy production and improving anti-inflammatory and antioxidative capacity. These findings provide compelling experimental support for our observation that NAIs effectively attenuate nicotine-induced oxidative damage in rat vasculature.

Nicotine activates the MAPK signaling pathway via α7nAChR, contributing to metabolic dysfunction [34]. Furthermore, it directly promotes the secretion of pro-inflammatory cytokines (IL-6 and TNF-α) in perivascular adipose tissue and enhances MAPK-mediated upregulation of adhesion molecules (Icam1 and Vcam1), thereby exacerbating inflammation [35]. Environmental epidemiological studies indicate that NAIs modulate oxidative and inflammatory responses while suppressing histidine, arginine, and proline metabolic pathways [36]. Reduced histidine levels correlate with diminished inflammation and oxidative stress in acute lung injury models [18], whereas decreased arginine and proline reflect enhanced anti-inflammatory capacity via endogenous NO production and reduced Ca^2+^ complexation [37]. Our findings demonstrate that nicotine binding to α7nAChR triggers intracellular signaling, inducing JNK/JUN phosphorylation and elevating JUN transcription. NAIs primarily counteract these effects by attenuating JNK/JUN phosphorylation. RNA-seq analysis identified FN1 and SPP1 as key differentially expressed genes that were upregulated by nicotine but downregulated upon NAI intervention. Mechanistically, nicotine–α7nAChR interaction activates the MAPK/AP1 pathway, inducing oxidative stress. Notably, nicotine exhibited cell-type-specific MAPK activation: it increased phospho-JNK and phospho-JUN in the thoracic aorta of male SHRs, whereas it predominantly elevated phospho-ERK in HAECs. Silencing FN1 or SPP1 did not alter MAPK/AP1 signaling, positioning these genes downstream of the pathway. Moreover, knockdown of AP1, FN1, or SPP1 had no effect on α7nAChR expression, confirming their dispensability in nicotine–receptor binding. However, AP1 silencing significantly reduced ROS levels and downregulated NOX2 and NOX4 expression, underscoring its critical role in oxidative stress regulation.

Fibulin, encoded by the FN1 gene, is a multifunctional extracellular matrix (ECM) component with distinct structural and functional properties. It plays a crucial role in various pathophysiological processes, including embryonic development, wound healing, and tissue remodeling [38]. Integrin-α5 (ITGA5), the primary ligand of FN1, mediates cytoskeletal remodeling, cell migration, apoptosis, and signal transduction through ECM interactions [39]. Emerging evidence suggests that AP1 activity influences ECM dynamics, with c-Jun—a key AP1 subunit—being upregulated in fibrotic diseases and forming a positive feedback loop with AKT (serine/threonine kinase) signaling [40]. While most studies on SPP1 have focused on its role in cancers (e.g., hepatocellular, colorectal, and cervical carcinomas) [41], recent work by Freiholtz et al. [42] identified SPP1 as a critical mediator of epithelial–mesenchymal transition (EMT) in degenerative ascending aortic aneurysms. In this context, SPP1 expression in the aortic intima–media correlated with inflammatory markers, macrophage infiltration, and aortic dilation, with E26 transcription factor-1 (ETS1) acting as a transcriptional regulator. Notably, the regulatory relationships between AP1 and FN1/SPP1 remain poorly understood. Our findings address this gap by demonstrating that FN1 is predominantly regulated by FOS, whereas SPP1 is primarily controlled by AP1.

Despite its findings, this study has several limitations that should be acknowledged. First, while animal models provide mechanistic insights, they cannot fully recapitulate the complex interplay between smoking, NAI intervention, and vascular endothelial dysfunction in hypertensive humans. Thus, future population-based studies are needed to validate our results—for instance, by examining the association between plasma nicotine levels, blood pressure, and endothelial dysfunction biomarkers in hypertensive smokers with varying exposure levels. Second, the absence of nicotine exposure experiments in WKY rats limits our ability to assess whether nicotine influences blood pressure and endothelial function in non-hypertensive conditions. Third, technical constraints prevented direct NAI exposure in vitro. Instead, we used gene knockdown approaches targeting key NAI-related mediators to indirectly evaluate their protective effects against nicotine-induced endothelial dysfunction. Although our data suggest that NAIs attenuate endothelial impairment by modulating AP1-mediated FN1 and SPP1 expression, due to the possible limitation of the small animal sample size, the precise molecular mechanisms require further elucidation. Lastly, while we focused on AP1, FN1, and SPP1 in nicotine-induced endothelial dysfunction, the broader regulatory network—including their roles in cytoskeletal signaling, focal adhesion dynamics, and ECM–receptor interactions—remains unexplored. Future studies should systematically investigate how AP1-dependent pathways influence vascular remodeling, which may uncover novel therapeutic targets for smoking-related endothelial injury.

In summary, this study elucidates the detrimental effects of nicotine on vascular endothelial oxidative damage and, for the first time, reveals that NAIs mitigate nicotine-induced endothelial dysfunction and SBP elevation in SHR. Mechanistically, NAIs downregulate the expression of FN1 and SPP1—key mediators of endothelial damage—via the suppression of AP1. Our findings identify FN1 and SPP1 as pivotal molecular targets linking endothelial dysfunction to hypertension, offering novel therapeutic insights for the prevention and treatment of cardiovascular diseases associated with nicotine exposure.

## Data Availability

The datasets generated and/or analyzed during the current study are available from the corresponding author upon reasonable request.

## Supplementary Materials

The following supporting information can be downloaded at https://www.mdpi.com/article/10.3390/antiox14070859/s1: Figure S1: Cell viability assay (*n* = 6); Table S1: siRNA primer sequences; Table S2: Primer sequences for rats; Table S3: Primer sequences for HAECs; Table S4: Fn1 and Spp1 primer sequences; Table S5: Prediction of AP1 binding site of Fn1 gene promoter region; Table S6: Prediction of AP1 binding site of Lamc3 gene promoter region; Table S7: Prediction of the AP1 binding site of the Vcam1 gene promoter region; Table S8: Prediction of the AP1 binding site of the Icam1 gene promoter region; Table S9: Prediction of the AP1 binding site of the Spp1 gene promoter region.

## Abbreviations

The following abbreviations are used in this manuscript:

nAChRs	Nicotinic Acetylcholine Receptors
ROS	Reactive Oxygen Species
NOX	NADPH Oxidases
VECs	Vascular Endothelial Cells
NO	Nitric Oxide
AP1	Activator Protein-1
MAPK	Mitogen-Activated Protein Kinase
ERK	Extracellular Signal-Regulated Kinases
JNK	c-Jun N-terminal Kinases
p38 MAPK	Stress-Activated Protein Kinases
MMP-9	Matrix Metalloproteinase-9
NAIs	Negative Air Ions
SOD	Superoxide Dismutase
SHR	Spontaneously Hypertensive Rat
Fn1	Fibronectin 1
Spp1	Secreted Phosphoprotein 1
WKY	Wistar-Kyoto
SBP	Systolic Blood Pressure
HAECs	Human Aortic Endothelial Cells
8-OHdG	8-Hydroxylated Deoxyguanosine
ECM	Extracellular Matrix
ITGA5	Integrin-α5
EMT	Epithelial–Mesenchymal Transition
ETS1	E26 Transcription Factor-1
