# Supplementary material for: Negative Air Ions Attenuate Nicotine-Induced Vascular Endothelial Dysfunction by Suppressing AP1-Mediated FN1 and SPP1
Source: Antioxidants (Basel). 2025 Jul 14;14(7):859. doi: 10.3390/antiox14070859 (PMC12292000; doi:10.3390/antiox14070859)
Supplement: Supplementary file 1 [file antioxidants-14-00859-s001.zip › antioxidants-3661556-supplementary.pdf]

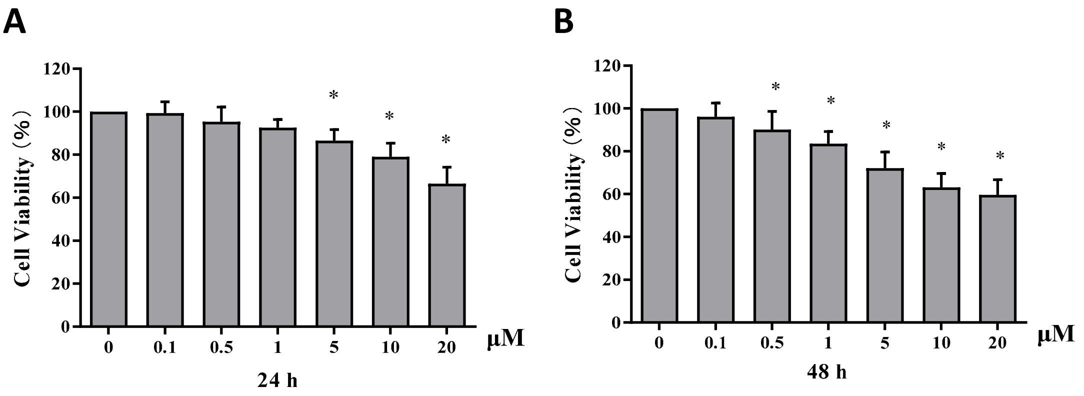

Figure S1 Cell viability assay (n=6). (A) HAECs were treated with 0, 0.1, 0.5, 1, 5, 10 and 20 μM of nicotine for 24 h. (B) HAECs were treated with 0, 0.1, 0.5, 1, 5, 10 and 20 μM of nicotine for 48 h. Compared to 0 μM, **P*<0.05.

Table S1 siRNA primer sequences

Genes	primer sequences (5' to 3')	Gene Accession Number
si-FN1	GAGCGTCCTAAAGACTCCATGATCT	NM_212482
si-SPP1	CCAGGACCTGAACGCGCCTTCTGAT	NM_000582.2
si-FOS	CCTGTCAACGCGCAGGACTTCTGCA	NM_005252
si-JUN	CACGTTAACAGTGGGTGCCAACTCA	NM_002228

Table S2 Primer sequences for rats

Genes	Forward (5' to 3')	Reverse (5' to 3')	Gene Accession Number
Fn1	CCA TCA CGC CGA GCA TTC T	TCT CCT CTC TGC CAT TAA CAG C	NM_019143.2
Lamc3	ACA CGA AGA GAA AGA CCA AGC A	TCT GGG AGT TGG AGG AGA GC	NM_001107830.1
Vcam1	AAC GCT CGC TCA GAT TGG A	TTC ACC TTC CCA TTT AGT GGA CT	NM_012889.2
Icam1	GAG ACT AAC TGG ATG AAA GAC GAA C	CAA TGT CGC TCA GCT TGA AGA	NM_012967.1
Spp1	TGA GGC TAT CAA GGT CAT CCC	TGT GTT TCC ACG CTT GGT TC	NM_012881.2
ß-actin	ATG GAA GAA GAA ATC GCC GC	ACA CGC AGC TCG TTG TAG AA	NM_007393.5

Table S3 Primer sequences for HAEC

Genes	Forward (5' to 3')	Reverse (5' to 3')	Gene Accession Number
FN1	CCAACCTTTACAGACCTATCC	GTCAAAGCGAGTCACTTCTTG	NM_212482
SPP1	GTACCCTGATGCTACAGACGAGG	CTCGTTTCATAACTGTCCTTCCC	NM_000582.2
GAPDH	CATCATCCCTGCCTCTACTGG	GTGGGTGTCGCTGTTGAAGTC	NM_001256799.2

Table S4 *Fn1* and *Spp1* primer sequences

Genes	primer sequences (5' to 3')		Tm	CG%	product length	Gene Accession Number
FN1	sense	TGATGAATGGTGCTAGGCTTC	57.8	47.6	242	NM_212482
	antisense	TTCAATGTCACTACTTTGGTGGAC	58.7	41.7		

SPP1	sense	CCTCTGTTCAAAGAGATTGCCT	58.0	45.5	179	NM_000582.2
	antisense	CTGGGAGTTTATCAAGCCATG	57.4	46.7		

Table S5 Prediction of AP1 binding site of Fn1 gene promoter region

Score	Relative score	Start	End	Strand	Predicted sequence
7.709	0.856	1161	1169	+	GTGCTTCAT
7.672	0.856	564	572	-	GTTAATCAC
7.453	0.851	1055	1063	+	ATGAGTCCC
5.505	0.813	916	924	-	TTAAGTCAT
5.472	0.812	925	933	-	GTGTTTCAC
5.231	0.807	1055	1063	-	GGGACTCAT
4.954	0.802	1209	1217	+	GTAAATCAT

Table S6 Prediction of AP1 binding site of Lamc3 gene promoter region

Score	Relative score	Start	End	Strand	Predicted sequence
8.963	0.881	1342	1350	-	GTGTGTCAC
8.605	0.874	1869	1877	-	ATGAGTTAT
7.263	0.847	1869	1877	+	ATAACTCAT
6.277	0.828	1256	1264	+	GGGAGTCAC
6.275	0.828	573	581	-	GTGAGCCAC
5.749	0.817	1361	1369	+	ATTGGTCAC
5.670	0.816	1051	1059	-	CTGCCTCAG
5.096	0.804	1483	1491	+	GTTCATCAC
4.923	0.801	573	581	+	GTGGCTCAC

Table S7 Prediction of AP1 binding site of Vcam1 gene promoter region

Score	Relative score	Start	End	Strand	Predicted sequence
9.293	0.888	881	889	-	ATTCGTCAC
8.034	0.863	1463	1471	+	ATTAGTAAT
6.843	0.839	1644	1652	+	TTGCCTCAT
6.812	0.839	1548	1556	-	ATGTGTCAA
6.650	0.835	415	423	-	ATTACTCAA
6.629	0.835	415	423	+	TTGAGTAAT
6.274	0.828	825	833	+	GTGAGTCCC
6.145	0.825	1463	1471	-	ATTACTAAT
6.134	0.825	1758	1766	+	ATAAATCAT
5.279	0.808	914	922	+	ATGAATCCT
5.121	0.805	789	797	-	CGGAGTCAC
5.114	0.805	1758	1766	-	ATGATTTAT
4.916	0.801	1240	1248	-	TTGAGTCTT

Table S8 Prediction of AP1 binding site of Icam1 gene promoter region

Score	Relative score	Start	End	Strand	Predicted sequence

7.311	0.848	1341	1349	-	CTGCCTCAC
7.160	0.845	1219	1227	-	GTTACTCAG
7.097	0.844	1219	1227	+	CTGAGTAAC
7.069	0.844	643	651	+	GTTCCTCAT
6.667	0.836	706	714	-	ATAAGTCAG
6.275	0.828	1696	1704	+	GTGAGCCAC
6.275	0.828	1969	1977	-	GTGAGCCAC
5.670	0.816	402	410	+	CTGCCTCAG
5.670	0.816	1556	1564	+	CTGCCTCAG
5.501	0.813	1858	1866	-	CTGAGACAC
5.433	0.811	1858	1866	+	GTGTCTCAG
5.426	0.811	1769	1777	+	CTGAGTTAC
4.922	0.801	1696	1704	-	GTGGCTCAC
4.922	0.801	1969	1977	+	GTGGCTCAC

Table S9 Prediction of AP1 binding site of Spp1 gene promoter region

Score	Relative score	Start	End	Strand	Predicted sequence
8.290	0.868	178	186	+	TTGAATCAT
7.808	0.858	1319	1327	-	CTGTGTCAC
7.340	0.849	1919	1927	-	TTGTGTCAT
7.289	0.848	178	186	-	ATGATTCAA
6.0551	0.824	596	604	-	ATTAGTCCT
5.344	0.809	113	121	+	ATGGTTCAT
5.280	0.808	113	121	-	ATGAACCAT
5.254	0.808	654	662	+	ATTTTTCAT
5.187	0.806	1017	1025	-	ATGCATCAA
5.128	0.805	667	675	-	GTGTGTAAT
